# Identification of the mechanism for dehalorespiration of monofluoroacetate in the phylum Synergistota

**DOI:** 10.5713/ab.23.0351

**Published:** 2023-12-29

**Authors:** Lex E. X. Leong, Stuart E. Denman, Seungha Kang, Stanislas Mondot, Philip Hugenholtz, Chris S. McSweeney

**Affiliations:** 1 CSIRO Agriculture and Food, St Lucia 4067, Queensland Australia; 3 Micalis Institute, INRA, AgroParisTech, University Paris-Saclay, 78350 Jouy-en-Josas, France; 4 Australian Centre for Ecogenomics, School of Chemistry and Molecular Bioscience, the University of Queensland, St Lucia, 4072 Queensland Australia

**Keywords:** Cloacibacillus, Dehalorespiration, Monofluoroacetate, Pyramidobacter, Rumen, Synergistota

## Abstract

**Objective:**

Monofluoroacetate (MFA) is a potent toxin that blocks ATP production via the Krebs cycle and causes acute toxicity in ruminants consuming MFA-containing plants. The rumen bacterium, *Cloacibacillus porcorum* strain MFA1 belongs to the phylum Synergistota and can produce fluoride and acetate from MFA as the end-products of dehalorespiration. The aim of this study was to identify the genomic basis for the metabolism of MFA by this bacterium.

**Methods:**

A draft genome sequence for *C. porcorum* strain MFA1 was assembled and quantitative transcriptomic analysis was performed thus highlighting a candidate operon encoding four proteins that are responsible for the carbon-fluorine bond cleavage. Comparative genome analysis of this operon was undertaken with three other species of closely related Synergistota bacteria.

**Results:**

Two of the genes in this operon are related to the substrate-binding components of the glycine reductase protein B (GrdB) complex. Glycine shares a similar structure to MFA suggesting a role for these proteins in binding MFA. The remaining two genes in the operon, an antiporter family protein and an oxidoreductase belonging to the radical S-adenosyl methionine superfamily, are hypothesised to transport and activate the GrdB-like protein respectively. Similar operons were identified in a small number of other Synergistota bacteria including type strains of *Cloacibacillus porcorum*, *C. evryensis*, and *Pyramidobacter piscolens*, suggesting lateral transfer of the operon as these genera belong to separate families. We confirmed that all three species can degrade MFA, however, substrate degradation in *P. piscolens* was notably reduced compared to *Cloacibacillus* isolates possibly reflecting the loss of the oxidoreductase and antiporter in the *P. piscolens* operon.

**Conclusion:**

Identification of this unusual anaerobic fluoroacetate metabolism extends the known substrates for dehalorespiration and indicates the potential for substrate plasticity in amino acid-reducing enzymes to include xenobiotics.

## INTRODUCTION

Fluorinated organic compounds (FOCs) are potentially harmful, persist in the environment, bioaccumulate and are globally distributed [1]. The environmental persistence of FOCs is due to the stability of the high-energy carbon–fluorine bond, which renders them largely resistant to microbial degradation and metabolism by animals. In the case of monofluoroacetate (MFA), animals metabolise the compound to fluorocitrate that inhibits the aconitase enzyme in the tricarboxylic acid cycle, thus disrupting cellular respiration and ATP production [2]. Aerobic and anaerobic bacteria utilise halogenated compounds including MFA as an energy source through hydrolytic and reductive mechanisms respectively [3–10]. Compared with aerobic degradation of MFA, there is a paucity of studies on the anaerobic degradation of this fluorinated compound. Recently, a bacterium capable of utilising MFA as an electron acceptor in a similar mechanism as other reductive dehalogenating bacteria was isolated from a bovine rumen [7,10]. This bacterium, *Cloacibacillus porcorum* strain MFA1 belongs to the bacterial phylum Synergistota and is capable of producing fluoride and acetate from MFA as the end-products of dehalorespiration [7,10]. Here, through comparative genomics and transcriptomics, we identify the operon responsible for this activity in strain MFA1 and other members of the Synergistota.

## MATERIALS AND METHODS

### Whole genome sequencing of *C. porcorum* strain MFA1

The draft genome of strain *C. porcorum* strain MFA1 was sequenced using a combined approach of 454 GS FLX Titanium shotgun sequencing (Roche Diagnostics, Branford, CT, USA), and 454 pair-end sequencing. *De novo* assembly was generated by Newbler assembler 2.0.00.20 (454 Life Sciences, Branford, CT, USA). The assembly was uploaded onto Integrated Microbial Genomes Expert Review (IMG/ER, https://img.jgi.doe.gov/cgi-bin/er/main.cgi) [11] annotation pipeline for gene annotation. The gene prediction was carried out using GeneMark [12], and Pfam, InterPro, COGs assignments were carried out to annotate genes. The annotated genome is deposited in the JGI IMG database accession number 2501651219.

Genome sequences of other Synergistota bacteria were retrieved from NCBI RefSeq FTP database (ftp://ftp.ncbi.nlm.nih.gov/genomes/refseq/bacteria/). Synteny of three *Cloacibacillus* species was determined using MUMmer program available at the IMG system. Homologous genes of the genomes of interest were identified by bidirectional best hit search using BLASTP with an E-value cutoff >1.0E^−5^. Multiple sequence alignments was performed using ClustalW [13], and consensus motives were identified in comparison with proteins experimentally validated from UniProtB database [14].

Candidate bacterial selenocysteine insertion sequence (SECIS) elements were detected using the online bSECISearch program (http://gladyshevlab.org/bSECISearch/) with default settings [15]. The secondary RNA structure of the SECIS elements were modelled using the online Vienna RNAfold program (http://rna.tbi.univie.ac.at/cgi-bin/RNAfold.cgi) [16] under the minimum free energy fold algorithm [17]. TMHMM Server 2.0, a membrane protein topology prediction method based on a hidden Markov model was used for *in silico* examination of transmembrane helices [18].

### RNA sequencing

*C. porcorum* strain MFA1 was cultivated under strict anaerobic conditions to mid-log phase (approximately an OD600 of 0.4) in 10 mL culture modified from previously [10] to contain an increase in yeast extract for improved growth of either 4% (w/v) yeast extract, or 0.8% (w/v) yeast extract and 20 mM MFA (sodium monofluoroacetate; ABCR GmbH & Co., KG Karlsruhe, Germany) [7,10]. The cultures were grown in triplicate, and pooled prior to total RNA extraction according to the method described previously [19]. Briefly, cells were pelleted after adding one-fifth volume of 5% (v/v) phenol:ethanol (pH4.3) to preserve the RNA content. The cell pellets were resuspended in 300 μL of 100 mM TE (Trisethylenediaminetetraacetic acid) buffer, pH 6. A further 400 μL of phenol:chloroform (1:1, pH4.3)(Sigma-Aldrich, St. Louis, MO, USA), 100 μL of 10% (w/v) sodium dodecyl sulfate and approximately 200 mg of diethylpyrocarbonate treated silica/zirconium beads (1:1 mixture of 0.1 mm and 1.0 mm in diameter; Biospec Scientific, Bartlesville, OK, USA) were added to the suspension. Samples were bead-beaten for 3×30 s at 4,000 RPM with a MO-BIO Powerlyzer 24 (MO-BIO Laboratories, Inc., Carlsbad, CA, USA). The homogenised suspension was then centrifuged for 2 min at 14,000×g. The aqueous phase was combined and mixed with 500 μL RLT buffer of the RNeasy Protect Bacteria Mini Kit (Qiagen, Hilden, Germany), before recovering RNA following the protocol from the RNeasy Mini kit.

Bacterial ribosomal RNAs were depleted using MICROB Express bacterial mRNA enrichment kit (Life Technologies, Carlsbad, CA, USA) following the manufacturer’s instructions. The enriched mRNA was fragmented using a modified method of the zinc-mediated RNA fragmentation [20]. Briefly, the mRNA was incubated with 10 mM ZnSO_4_ in 10 mM Tris-HCl (pH 7.3) at 70°C for 45 s. The reaction was stopped with 50 mM EDTA in 10 mM Tris-HCl (pH 7.3). The fragmented RNA was then purified by ethanol precipitation, and complementary DNA was synthesised using SuperScript double stranded cDNA synthesis system (Life Technologies, USA) with random hexamer primers. High-throughput cDNA sequencing was performed using Roche 454 shotgun sequencing on a quarter plate by a Roche 454 GS-FLX Titanium as per manufacturer’s instructions.

Quality-trimmed reads were aligned to strain MFA1 draft genome using Burrows-Wheeler Aligner [21]. Genome expression profiles were inferred using SAMtools [22]. Profiles were normalised by dividing each base position by the total number of bases mapped. Gene counts were determined using the maximum nucleotidic base coverage per gene. Gene counts normalisation was similar to the genome expression profiles. Finally, differential gene expression was performed according to the culture conditions, and candidate genes for MFA degradation were selected for further validation using quantitative real–time polymerase chain reaction (qRT-PCR) quantification.

qRT-PCR validation was performed using total RNAs from a second experiment of strain MFA1 cells in a similar growth phase and media. The primers for gene components of far operon (Supplementary Table S3) were designed using PRISE [23]. The expression levels of these genes were quantified using ViiA 7 Real-Time PCR system (Life Technologies, USA) with Platinum SYBR Green qPCR SuperMix-UDG as recommended by the manufacturer (Life Technologies, USA). The expression of the 16S rRNA gene in both treatments was used as the reference gene to normalise other gene expression using the 2^−ΔΔCT^ method [24].

### Inducibility and specificity of the candidate *far* operon using qRT-PCR

In order to determine the effect of glycine on the expression of the *far* operon, the candidate genes expressions for strain MFA1 cells in growth media with 20 mM glycine, or 20 mM MFA were compared to MFA1 expression in basal yeast extract medium [10]. The inducibility of the *far* operon by MFA was examined by introducing 5 mM MFA to a log phase *C. porcorum* strain MFA1 culture in basal growth medium. Strain MFA1 was subcultured in a growth medium without MFA twice before the induction study. Filter-sterilised sodium monofluoroacetate, or sterile anaerobic diluent was introduced to the culture in triplicate, respectively at log phase (after 12 hours incubation), and samples were collected at 0-, 1-, 3-, 5-, and 12-hours post MFA introduction. The gene expression of *far* candidate operon was measured using a similar qRT-PCR approach.

### Fluoroacetate degradation assay

Bacterial strains *C. porcorum* strain MFA1, *C. porcorum* strain CL-84, *C. evryensis* 158, DSM 19522, *Pyramidobacter piscolens* W5455, and *Synergistes jonesii* 78-1 were grown in the presence of 10 mM fluoroacetate and their ability to degrade fluoroacetate was through the detection of fluoride production using a Thermo Scientific fluoride ion-selective electrode (Thermo Fisher Scientific, Waltham, MA, USA), as described previously [7].

## RESULTS AND DISCUSSION

**Figure 1:**
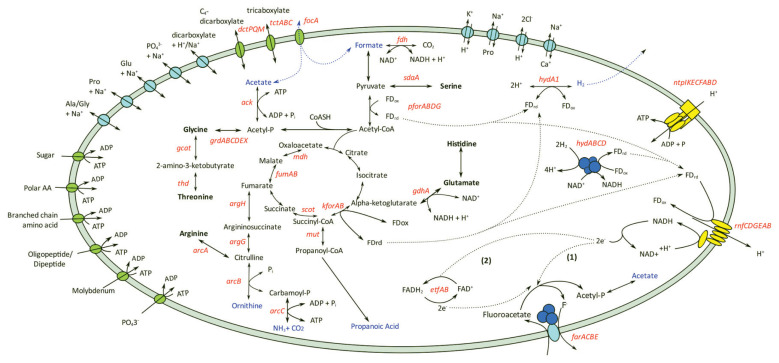
Energy conservation pathways and solute transport of *Cloacibacillus porcorum* strain MFA1. Amino acids utilised by strain MFA1 were shown in bold, and metabolite products were highlighted in blue. Genes identified from the genome were labelled in red. Genes annotated within amino acid metabolic pathways are argininosuccinate lyase (*argH*), argininosuccinate synthethase (*argG*), arginine deiminase (*arcA*), ornithine carbamoyltransferase (*arcB*), carbamate kinase (*arcC*), serine ammonia-lyase (*sdaA*), L-threonine 3-dehydrogenase (*thd*), glycine C-acetyltransferase (*gcat*), glycine reductase (*grdABCDEX*) and glutamate dehydrogenase (*gdhA*). Genes involved with energy or metabolic product formations are formate dehydrogenase (*fdh*), pyruvate:ferredoxin oxidoreductase (*pforABC*), succinyl-CoA:oxoacid transferase (*scot*), malate dehydrogenase (*mdh*), fumarase (*fumAB*), α-ketoglutarate:ferredoxin oxidoreductase (*kforAB*), methylmalonyl-CoA mutase (*mut*), acetate kinase (*ack*), electron-transferring flavoprotein (*etfAB*), iron-only hydrogenase (*hydA1*), NADH-dependent FeFe-hydrogenase (*hydABC*), “*Rhodobacter*-nitrogen fixation” (*rnfCDGEAB*) and archaeal/vacuolar-type H^+^-ATPase (*ntpABCDEFGHI*). Enzymatic reactions with secondary active transporters are shown in light blue, while other transporters including ATP-binding cassette (ABC) transporter, tripartite ATP-independent periplasmic (*dctPQM*), tripartite tricarboxylate transporter (*tctABC*) and formate transporter are shown in green. ABC transporters are indicated with an exchange of ATP with ADP.

The draft genome of *C. porcorum* strain MFA1 comprises 19 scaffolds with an estimated genome size of 3,525,432 bp, and 3,239 coding sequences. Strain MFA1 is highly syntenous with the genomes of both *Cloacibacillus porcorum* (type strain) and *C. evryensis* (Supplementary Figure S1; Supplementary Table S1). Thirteen percent of strain MFA1 genes encode amino acid transport and metabolism suggesting that this organism derives energy from exogenous amino acids and peptides [7,10,25]. Complete amino acid fermentation pathways detected in the *C. porcorum* strain MFA1 genome are involved in the catabolism of the following amino acids: arginine, asparagine, glutamate, glycine, histidine, lysine, serine and threonine (Figure 1) [7,10]. Metabolism of these amino acids contributes to the energy conservation in strain MFA1 through direct production of ATP via substrate level phosphorylation (glycine reductase mechanism), and through electron bifurcation with electron-bifurcating [FeFe]-hydrogenases (*hydABC*) and the proton-translocating ferredoxin:NAD^+^ oxidoreductase Rhodobacter nitrogen fixation complex (*rnfCDGEAB*; SFA1_28680-28730) (Figure 1).

**Figure 2:**
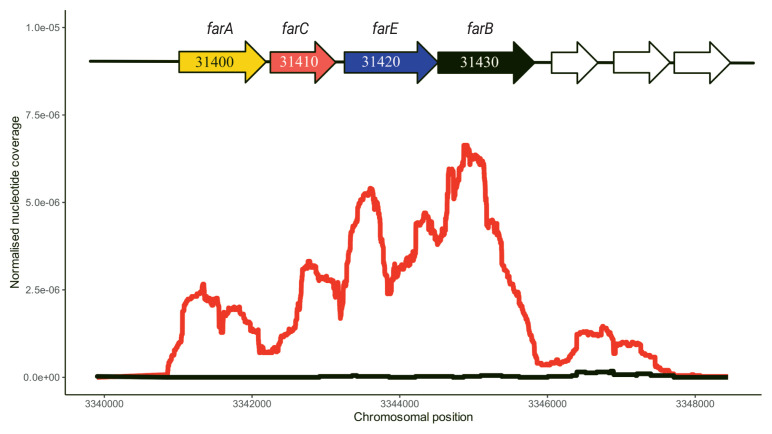
Expression profile of the candidate *farACEB* operon with number of mapped sequencing reads to the genes along the chromosome. Red line represents aligned reads to the chromosome in *C. porcorum* strain MFA1 cells with MFA, while black line represents aligned reads to the chromosome in *C. porcorum* strain MFA1 cells without MFA. MFA, monofluoroacetate.

*C. porcorum* strain MFA1 possesses two sets of operons encoding the subunits of archaeal/vacuolar-type H^+^ ATPase, but does not encode F-type ATPase. In the ATP synthesis mode, the A/V-type ATPases of strain MFA1 potentially harness energy from the transmembrane proton gradients generated by flavoproteins (Figure 1). In addition, strain MFA1 produces energy using MFA as a reducing agent for anaerobic respiration [7,10]. Transcriptomic analysis of strain MFA1 grown in media supplemented with MFA at mid-log growth phase led to the identification of an operon composed of four genes involved in the cleavage of the MFA carbon-fluorine bond (Figure 2). This observation was further verified using quantitative RT-PCR, indicating up-regulation of this operon by an average of 5,000 fold in cells supplemented with MFA (Supplementary Table S2). Furthermore, the same genes comprising the operon were induced following the introduction of MFA. Actively growing *C. porcorum* strain MFA1 cells in a non-MFA medium showed increased operon expression following 5 mM MFA introduction compared to cells at mid-log phase (300 fold after one hour, and 3,000 fold over five hours; Supplementary Figure S3).

The proposed *farACEB* (fluoroacetate reductase) operon consists of four genes encoding a secondary active transporter, an iron-sulphur oxidoreductase and two components of the glycine reductase (GR) substrate-specific protein complex B (Figure 2). The antiporter (*farA*; MFA1_31400) a member of the major facilitator superfamily belonging to the subfamily of oxalate/formate antiporters, which generate electrochemical potential differences to drive ATP synthesis [26], is likely to transport MFA in exchange for its product fluoride. An oxidoreductase (*farC*; MFA1_31410) belonging to the radical S-adenosyl methionine (SAM) superfamily may play a role in reactivation of the selenocysteine active residue in *farB* when it becomes oxidised to selenic acid [27]. The two remaining genes encode GR protein B complex homologues and are predicted to be involved in binding MFA thus allowing nucleophilic attack by the selenocysteine residue on the polar carbon-fluorine (C–F) bond (*farB*; MFA1__31430/MFA1_31440, and *farE*; MFA1_31420) [27].

**Figure 3:**
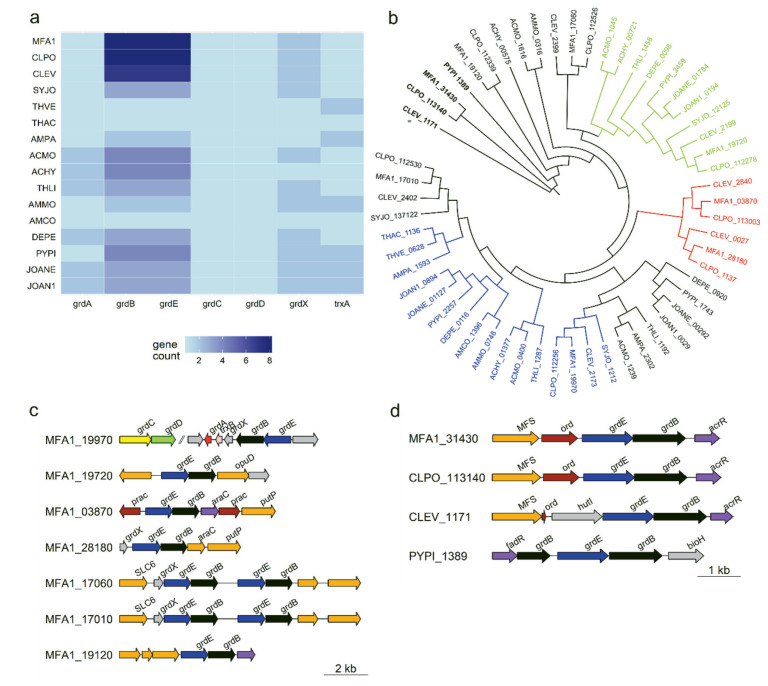
The evolution of glycine reductase protein B complex in Synergistota phylum. (a) Gene count of the components of glycine reductase complex indicating overabundance of *grdB* in *Cloacibacillus porcorum* strain MFA1 (MFA1) and *C. porcorum* strain CL-84 (CLPO) as compared to 14 other Synergistota bacteria: *Cloacibacillus evryensis* (CLEV), *Synergistes jonesii* (SYJO), *Thermanaerovibrio velox* (THVE), *Thermanaerovibrio acidaminovorans* (THAC), *Aminomonas paucivorans* (AMPA), *Acetomicrobium mobile* (ACMO), *Acetomicrobium hydrogeniformans* (ACHY), *Thermovirga lienii* (THLI), *Aminobacterium mobile* (AMMO), *Aminobacterium colombiense* (AMCO), *Dethiosulfovibrio peptidovorans* (DEPE), *Pyramidobacter piscolens* (PYPI), *Jonquetella anthropi* E3_33E1 (JOANE), and *Jonquetella anthropi* ADV126 (JOAN1). (b) Maximum-likelihood phylogenetic tree of *grdB* genes amongst the 15 Synergistota bacteria. Terminal node labels are represented by abbreviations consisting of bacterial identification and genomic loci. (c) Genetic map organisation of *Cloacibacillus porcorum* strain MFA1 gene clusters containing glycine reductase protein complex B. Genes involved in glycine reduction were individually highlighted: *grdA* (red), *grdB* (black), *grdE* (blue), *grdC* (yellow), *grdD* (green), *trxA* (pink) and *grdX* (grey); genes encoding for transporters were highlighted in orange (sodium:neurotransmitter/glycine symporter, SLC6; proline:sodium symporter, *putP*; betaine transporter, *opuD*; major facilitator superfamily, MFS), regulatory genes were shown in purple, and other genes with unknown functions were shown in brown. (d) Genetic map of gene clusters from genera *Cloacibacillus* and *Pyramidobacter* with close sequence similarity to *farB*. Other genes within the gene clusters consisted of imidazolonepropionase (*hutI*), alpha-beta hydrolase (*bioH*), transcription regulatory protein (*fadR* and *acrR*).

Many bacteria have multiple substrate-specific protein B complexes which feed into a common reductase system to produce acetyl-phosphate [27]. *Cloacibacillus porcorum* strain MFA1 has a greater diversity and representation of GR protein complex B genes relative to other genera in the Synergistota (Figure 3a). The glycine reductase protein B (*grdB*) and *grdE* genes generally cluster into substrate-specific functional groups. Strain MFA1 contains substrate specific proteins B for glycine, proline, glycine-betaine and sarcosine [28], as identifiable in close proximity with associated genes like betaine transporter *opuD* gene and proline racemase (Figure 3b). All eight hypothethical *grdB* genes from strain MFA1 are predicted to be selenocysteine-containing proteins due to the presence of a selenocysteine insertion sequence (SECIS), an RNA element that occurs downstream of the in-frame UGA codon (Supplementary Figure S4a). In addition to the SECIS element, strain MFA1 harbours all the genes required for the co-translational insertion of selenocysteine during selenoprotein biosynthesis and the selenocysteine-specific tRNA (tRNASec) (Supplementary Figure S4b), and has an absolute requirement for selenium in the growth media. Despite MFA having a similar chemical structure to glycine (Supplementary Figure S2), the *farACEB* operon was not highly upregulated in the presence of glycine (Supplementary Table S2) attesting to the substrate specificity of the MFA-specific GRB complex. In *C. porcorum* strain MFA1, the carboxymethyl-selenoether produced from the MFA-specific GR protein B complex is predicted to be transferred to the GR protein A and C components of the GR system. The connection between the MFA-specific GRB complex (*farEB*) with the GR protein A and C is indicated by upregulation of the *grdA* gene in the presence of MFA, contributing just over 1% of the total transcripts (data not shown).

Both of the MFA protein complex B genes show variance to the canonical GR protein B complex; *farB* lacks the conserved CxxC residues involved in protecting the active site Sec from oxidation [27], while *farE* does not contain the two conserved cysteines for autocatalytic processing and production of an N-terminal pyruvoyl group, and therefore is not a pro-protein, similar to the glycine-betaine *grdI* [29]. For the reduction of glycine and sarcosine, the carbon-nitrogen bond is polarised through the binding of the substrate and formation of a Schiff base with a carbonyl group close to the Sec to allow nucleophilic attack. A potential candidate for the unidentified carbonyl group is the serine in the conserved STUG motif of *grdB*, which would require modification to a formylglycine group similar to that for sulfatases [30,31]. Potentially, this function could be performed by the radical SAM (rSAM) found in the operon. However, its relevance to binding MFA would not be essential as no Schiff base can form, suggesting the preferred substrate would be another amine-containing compound.

Reduction mechanisms, such as glycine and MFA reduc tion that require a synergistic group of protein complexes can be strongly affected by the operon organisation and their regulatory effects [32,33]. Loss of the rSAM (*farC*) or transporter gene (*farA*) resulted in marked decreases in MFA degradation capability. The *far* operon of *C. evryensis* does not contain the rSAM, and MFA degradation was reduced by 40% (Supplementary Table S4). Neither the rSAM nor the transporter was evident in the *P. piscolens* genome which likely explained the observed 70% reduction in activity (Supplementary Table S4).

Gene duplication with subsequent sequence and functional divergence have been universally regarded as an important means for broadening the phenotypes and adaptive behaviour of bacteria [34,35]. While some members of the gene families may have been lost over time, the presence of duplicate genes are crucial for the organism’s adaptation to a range of specialised environmental niches [34,36]. The presence of actively transcribed *farB* and *farE* genes, homologous to the glycine reductase *grdB* and *grdE* genes, identifies the mechanism for anaerobic cleavage of the highly stable C–F bond. Promoting the activity of this protein complex in its native or a heterologous host may provide an avenue for the detoxification of MFA in anoxic environments.

In conclusion, we confirmed that three species belonging to the Synergistota, namely *C. porcorum*, *C. evryensis*, and *P. piscolens* can degrade MFA. However, substrate degradation in *P. piscolens* was notably reduced compared to the *Cloacibacillus* species possibly reflecting the loss of the oxidoreductase and antiporter in the *P. piscolens* operon. Identification of this unusual anaerobic fluoroacetate metabolism extends the known substrates for dehalorespiration and indicates the potential for substrate plasticity in amino acid-reducing enzymes to include xenobiotics.

## SUPPLEMENTARY MATERIAL

**Supplementary Table S1.** Genome average nucleotide identity similarity

**Supplementary Table S2.** Relative expression of the Sfa_31400-31440 operon in different substrates

**Supplementary Table S3.** Primers used in this study

**Supplementary Table S4.** In-vitro fluoroacetate degradation by Synergistota bacteria in a growth media containing 10 mM fluoroacetate

**Supplementary Figure S1.** Nucleotide synteny between Cloacibacillus porcorum str MFA1 with Cloacibacillus porcorum str CL-84(T) and C. evryensis.

**Supplementary Figure S2.** Chemical structures of fluoroacetate and glycine.

**Supplementary Figure S3.** Detected changes for the expression of three genes from the Sfa1_31400-31440 operon after introduction of 5 mM fluoroacetate to 12 hr old cultures of C. porcorum str MFA1.

**Supplementary Figure S4.** Glycine reductase protein complex B of C porcorum str MFA1.
