# Supplementary material for: Identification of the mechanism for dehalorespiration of monofluoroacetate in the phylum Synergistota
Source: Anim Biosci. 2023 Dec 29;37(2):396–403. doi: 10.5713/ab.23.0351 (PMC10838667; doi:10.5713/ab.23.0351)
Supplement: Supplementary file 1 [file ab-23-0351-Supplementary-Table-1.pdf]

Supplementary Table 1. Genome average nucleotide identity similarity

Genome1	Genome2	ANI1->2	ANI2->1	AF1->2	AF2->1
Cloacibacillus porcorum MFA1	*Cloacibacillus porcorum* CL-84	98.36	98.35	79.02	76.09
Cloacibacillus porcorum MFA1	*Cloacibacillus evryensis* 158, DSM 19522	84.41	84.42	61.12	60.22
Cloacibacillus porcorum MFA1	*Cloacibacillus* sp. An23	75.98	75.98	40.88	48.35
Cloacibacillus porcorum MFA1	*Pyramidobacter piscolens* W5455	67.69	67.72	6.60	8.63
Cloacibacillus porcorum CL-84	*Cloacibacillus evryensis* 158, DSM 19522	84.67	84.61	66.82	67.52
Cloacibacillus porcorum CL-84	*Cloacibacillus* sp. An23	75.98	75.93	43.43	52.61
Cloacibacillus porcorum CL-84	*Pyramidobacter piscolens* W5455	67.83	67.78	7.18	9.70
Cloacibacillus evryensis 158, DSM 19522	*Cloacibacillus* sp. An23	76.23	76.21	41.61	50.01
Cloacibacillus evryensis 158, DSM 19522	*Pyramidobacter piscolens* W5455	68.14	68.16	7.23	9.66
Cloacibacillus sp. An23	*Pyramidobacter piscolens* W5455	68.85	68.85	7.99	8.87
