# Supplementary material for: Identification of the mechanism for dehalorespiration of monofluoroacetate in the phylum Synergistota
Source: Anim Biosci. 2023 Dec 29;37(2):396–403. doi: 10.5713/ab.23.0351 (PMC10838667; doi:10.5713/ab.23.0351)
Supplement: Supplementary file 2 [file ab-23-0351-Supplementary-Table-2.pdf]

Supplementary Table 2. Relative expression of the Sfa_31400-31440 operon in different substrates.

**Substrate (20 mM)**	**OD_{600}**	**Fluoride production (mM)**	**Fold change**			
			**Sfa1_31400**	**Sfa1_31410**	**Sfa1_31420**	**Sfa1_31430**
Fluoroacetate	0.220	2.6 ± 0.1	5,181 ± 512	6,036 ± 640	4,256 ± 436	4,758 ± 500
Glycine	0.390	-	13 ± 4	17 ± 5	10 ± 3	10 ± 2

Fold change of samples against the controls in BYE medium were calculated using 2ΔΔCT method, with 16S rRNA as reference gene.
