# Supplementary material for: Identification of the mechanism for dehalorespiration of monofluoroacetate in the phylum Synergistota
Source: Anim Biosci. 2023 Dec 29;37(2):396–403. doi: 10.5713/ab.23.0351 (PMC10838667; doi:10.5713/ab.23.0351)
Supplement: Supplementary file 3 [file ab-23-0351-Supplementary-Table-3.pdf]

Supplementary Table 3. Primers used in this study.

**Locus Tag**	**Primer**	**Oligonucleotide sequence**	**Amplicon Size (bp)**
Sfa1_31400 (*farA*)	SFA1_31400_F	CGTGAGTCAGTGGCTGAAAAA	179
	SFA1_31400_R	CGCTTACCTCATACTGCTTCTT	
Sfa1_31410 (*farC*)	SFA1_31410_F	CAACGTCCTGGCCTTCAAAA	199
	SFA1_31410_R	AATCTGCGCGTTTCCGGATT	
Sfa1_31420 (*farE*)	SFA1_31420_F	CTTATGAAGCGTCACGGCAAA	182
	SFA1_31420_R	GCTGAATCAAGCTGACAGTG	
Sfa1_31430 (*farB*)	SFA1_31430_F	CCAGGCACGGATCTTTACAAAA	189
	SFA1_31430_R	AATGGCGTAATCAATGGCGAC	
16S_ rRNA	SFA1_16S_F	CCCTATGTCCAGTTGCTAACAA	160
	SFA1_16S_R	GGCTTTTAAGGATTCGCCAACT	
