# Supplementary material for: Identification of the mechanism for dehalorespiration of monofluoroacetate in the phylum Synergistota
Source: Anim Biosci. 2023 Dec 29;37(2):396–403. doi: 10.5713/ab.23.0351 (PMC10838667; doi:10.5713/ab.23.0351)
Supplement: Supplementary file 4 [file ab-23-0351-Supplementary-Table-4.pdf]

Supplementary Table 4. *In-vitro* fluoroacetate degradation by Synergistota bacteria in a growth media containing 10 mM fluoroacetate

**Bacteria**	**Fluoroacetate degraded (mM)**
Cloacibacillus porcorum str MFA1	10.0
Cloacibacillus porcorum str CL-84(T)	10.0
Cloacibacillus evryensis	6.0
Pyramidobacter piscolens	3.0
Synergistes jonesii	0
