# Supplementary material for: Identification of the mechanism for dehalorespiration of monofluoroacetate in the phylum Synergistota
Source: Anim Biosci. 2023 Dec 29;37(2):396–403. doi: 10.5713/ab.23.0351 (PMC10838667; doi:10.5713/ab.23.0351)
Supplement: Supplementary file 5 [file ab-23-0351-Supplementary-Fig-1.pdf]

Identification of the mechanism for dehalorespiration of monofluoroacetate in the phylum Synergistota

Lex E.X. Leong[1], https://orcid.org/0000-0002-5073-2272

Stuart E. Denman[1,*], https://orcid.org/0000-0002-9910-3709

Seungha Kang[1,#], https://orcid.org/0000-0003-0731-5876

Stanislas Mondot[2], https://orcid.org/0000-0003-3668-3168

Philip Hugenholtz[3], https://orcid.org/0000-0001-5386-7925

Chris S. McSweeney[1], https://orcid.org/0000-0002-0114-5151

*Corresponding Author: Stuart E. Denman, Tel: +61-07-32142273, E-Mail: stuart.denman@csiro.au

[1] CSIRO Agriculture and Food, St Lucia 4067, Queensland Australia

[2] Micalis Institute, INRA, AgroParisTech, University Paris-Saclay, 78350 Jouy-en-Josas France

[3] Australian Centre for Ecogenomics, School of Chemistry and Molecular Bioscience, the University of Queensland, St Lucia, 4072 Queensland Australia

[#] Current address: The University of Queensland Frazer Institute, Faculty of Medicine, University of Queensland, Brisbane, Queensland, Australia

Supplementary Tables and Figures

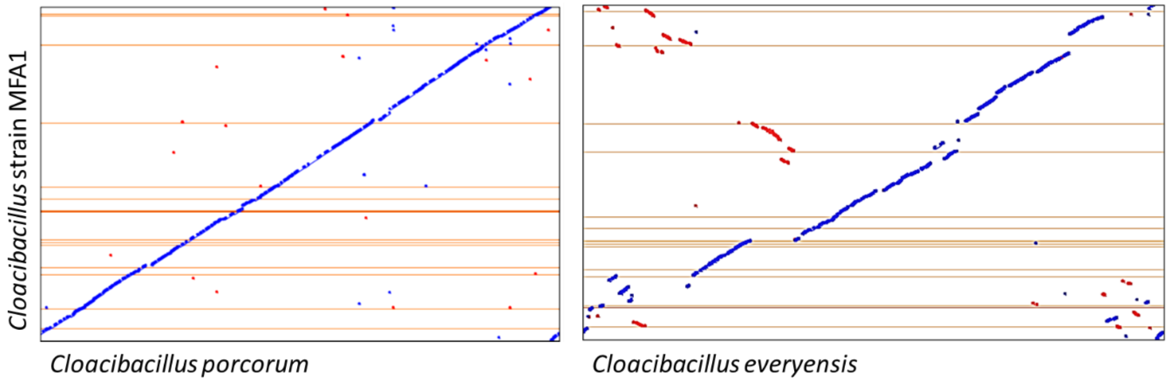

Supplementary Figure 1.

Nucleotide synteny between *Cloacibacillus porcorum* str MFA1 with *Cloacibacillus porcorum* str CL-84(T) and *C. evryensis*. Dots represent matches between the chromosome sequences: matches in the same orientation in both chromosomes are shown in blue, and matches with reverse orientation are shown in red.
