# Supplementary figures and images for: Identification of the mechanism for dehalorespiration of monofluoroacetate in the phylum Synergistota
Source: Anim Biosci. 2023 Dec 29;37(2):396–403. doi: 10.5713/ab.23.0351 (PMC10838667; doi:10.5713/ab.23.0351)

29

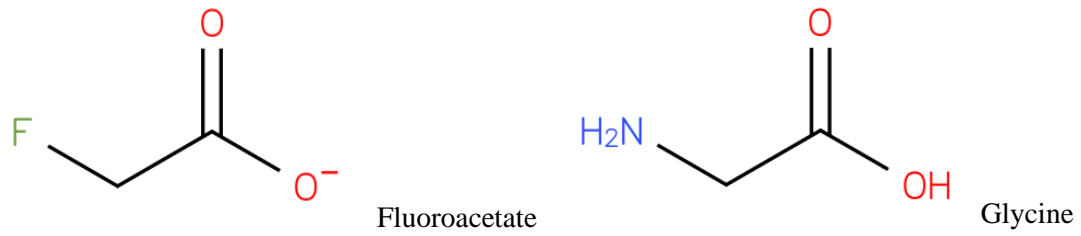

30

### 31 **Supplementary Figure 2.**

32 Chemical structures of fluoroacetate and glycine.

33

34

Supplement: Supplementary file 6 [file ab-23-0351-Supplementary-Fig-2.pdf]
