# Supplementary material for: Identification of the mechanism for dehalorespiration of monofluoroacetate in the phylum Synergistota
Source: Anim Biosci. 2023 Dec 29;37(2):396–403. doi: 10.5713/ab.23.0351 (PMC10838667; doi:10.5713/ab.23.0351)
Supplement: Supplementary file 7 [file ab-23-0351-Supplementary-Fig-3.pdf]

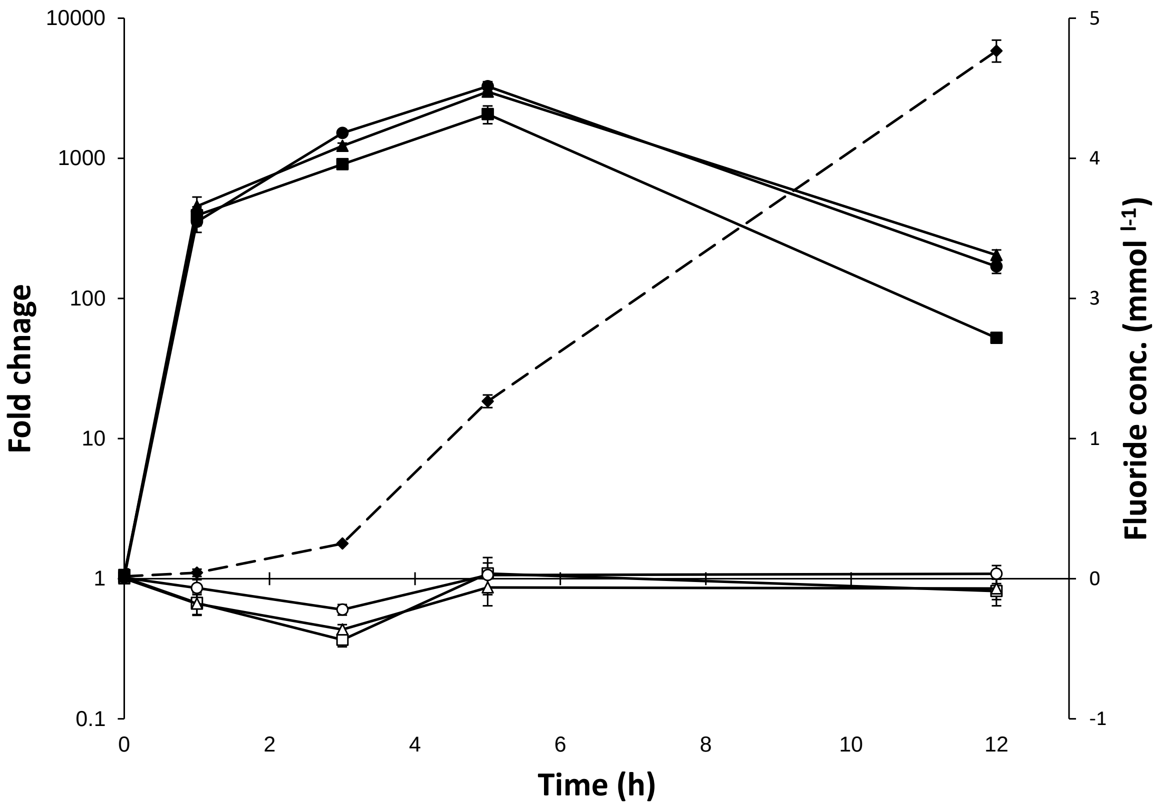

Supplementary Figure 3.

Detected changes for the expression of three genes from the Sfa1_31400-31440 operon after introduction of 5 mM fluoroacetate to 12 hr old cultures of *C. porcorum* str MFA1. Cultures pulsed with 5 mM fluoroacetate (solid markers), and anaerobic diluent (open markers) in triplicate. Square (■) represents the Sfa1_31410 (*farC*), triangle (▲) represents Sfa1_31420 (*farE*) and circle represents (●) Sfa1_31430/31440 (*farB*). Relative fold change of gene expression against time 0 hour is shown on the primary *y*-axis with $\log_{10}$ scale, fluoride concentration from defluorination activity (dashed line) is shown on the secondary *y*-axis.
