# Supplementary material for: Identification of the mechanism for dehalorespiration of monofluoroacetate in the phylum Synergistota
Source: Anim Biosci. 2023 Dec 29;37(2):396–403. doi: 10.5713/ab.23.0351 (PMC10838667; doi:10.5713/ab.23.0351)
Supplement: Supplementary file 8 [file ab-23-0351-Supplementary-Fig-4.pdf]

a

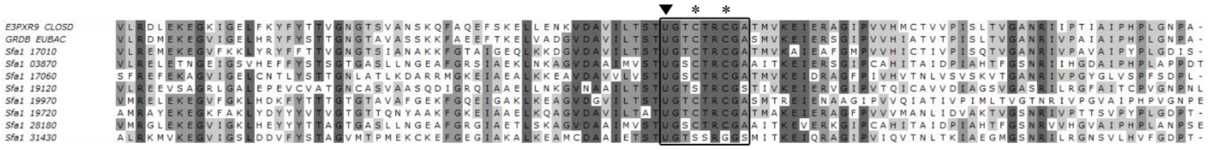

b

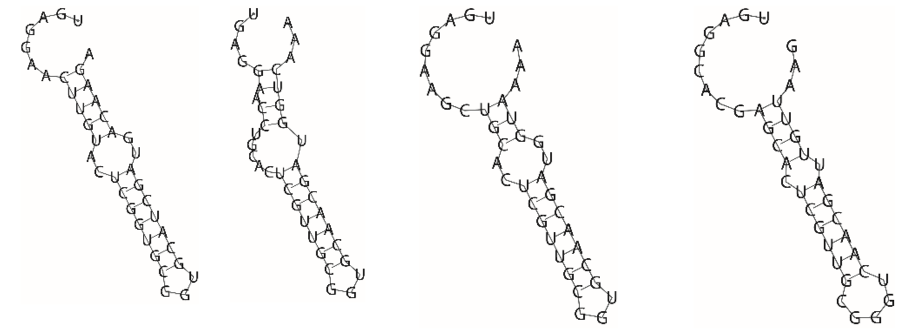

Locus Tag	Sfa1_19970	Sfa1_17010	Sfa1_17060	Sfa1_19120
MFE (kcal/mol)	-14.05	-8.99	-10.76	-8.91
Frequency (%)	92	38	79	72

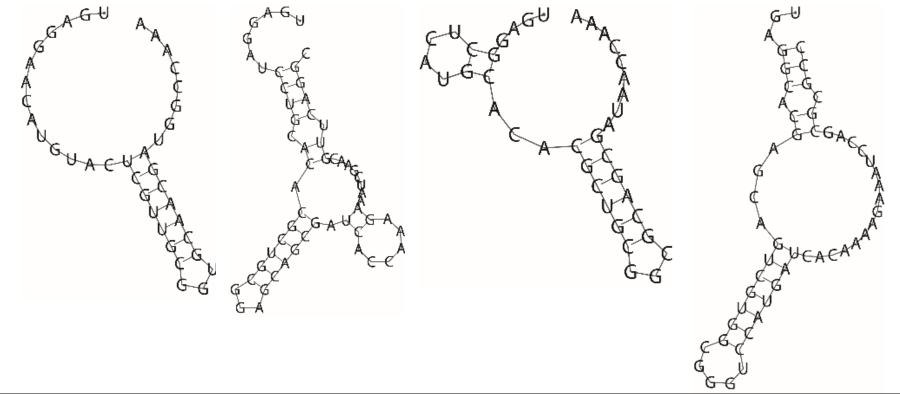

Locus Tag	Sfa1_19720	Sfa1_03870	Sfa1_28180	Sfa1_31430
MFE (kcal/mol)	-8.5	-12.1	-10.82	-10.38
Frequency (%)	72	64	51	87

Supplementary Figure 4.

Glycine reductase protein complex B of *C porcorum* str MFA1. (a)

Multiple sequence alignment of genes annotated as glycine reductase protein complex B of

strain MFA1 with glycine reductase protein complex B of *Clostridium sticklandii* (E3PXR9_CLOSD) and *Eubacterium acidaminophilum* (GRDB_EUBAC). Boxed amino acid sequences represent the region of the selenocysteine insertion sequence (SECIS) element, selenocysteine catalytic site is marked with an inverted triangle (▼), and the two conserved Cys residues involved in protecting the Sec site from oxidation are marked with asterisks (*). (b) Predicted RNA secondary structures of SECIS elements in glycine reductase protein Bs of MFA1. The minimal free energy (MFE) secondary structures were constructed using Vienna RNA fold as described in Materials and Methods. The free energy of the thermodynamic ensemble in kcal/mol and frequency of the MFE structure was used to predict the robustness of the RNA secondary structures.
